# Biochemical and structural characterisation of the second oxidative crosslinking step during the biosynthesis of the glycopeptide antibiotic A47934

**DOI:** 10.3762/bjoc.12.284

**Published:** 2016-12-27

**Authors:** Veronika Ulrich, Clara Brieke, Max J Cryle

**Affiliations:** 1 Department of Biomolecular Mechanisms, Max Planck Institute for Medical Research, Jahnstrasse 29, 69120 Heidelberg, Germany; 2 EMBL Australia, Monash University, Clayton, Victoria 3800, Australia; 3 The Monash Biomedicine Discovery Institute, Department of Biochemistry and Molecular Biology and ARC Centre of Excellence in Advanced Molecular Imaging, Monash University, Clayton, Victoria 3800, Australia

**Keywords:** crystal structure, cytochrome P450, glycopeptide antibiotic, peptide, phenolic coupling

## Abstract

The chemical complexity and biological activity of the glycopeptide antibiotics (GPAs) stems from their unique crosslinked structure, which is generated by the actions of cytochrome P450 (Oxy) enzymes that affect the crosslinking of aromatic side chains of amino acid residues contained within the GPA heptapeptide precursor. Given the crucial role peptide cyclisation plays in GPA activity, the characterisation of this process is of great importance in understanding the biosynthesis of these important antibiotics. Here, we report the cyclisation activity and crystal structure of StaF, the D-*O*-E ring forming Oxy enzyme from A47934 biosynthesis. Our results show that the specificity of StaF is reduced when compared to Oxy enzymes catalysing C-*O*-D ring formation and that this activity relies on interactions with the non-ribosomal peptide synthetase via the X-domain. Despite the interaction of StaF with the A47934 X-domain being weaker than for the preceding Oxy enzyme StaH, StaF retains higher levels of in vitro activity: we postulate that this is due to the ability of the StaF/X-domain complex to allow substrate reorganisation after initial complex formation has occurred. These results highlight the importance of testing different peptide/protein carrier constructs for in vitro GPA cyclisation assays and show that different Oxy homologues can display significantly different catalytic propensities despite their overall similarities.

## Introduction

**Figure 1:**
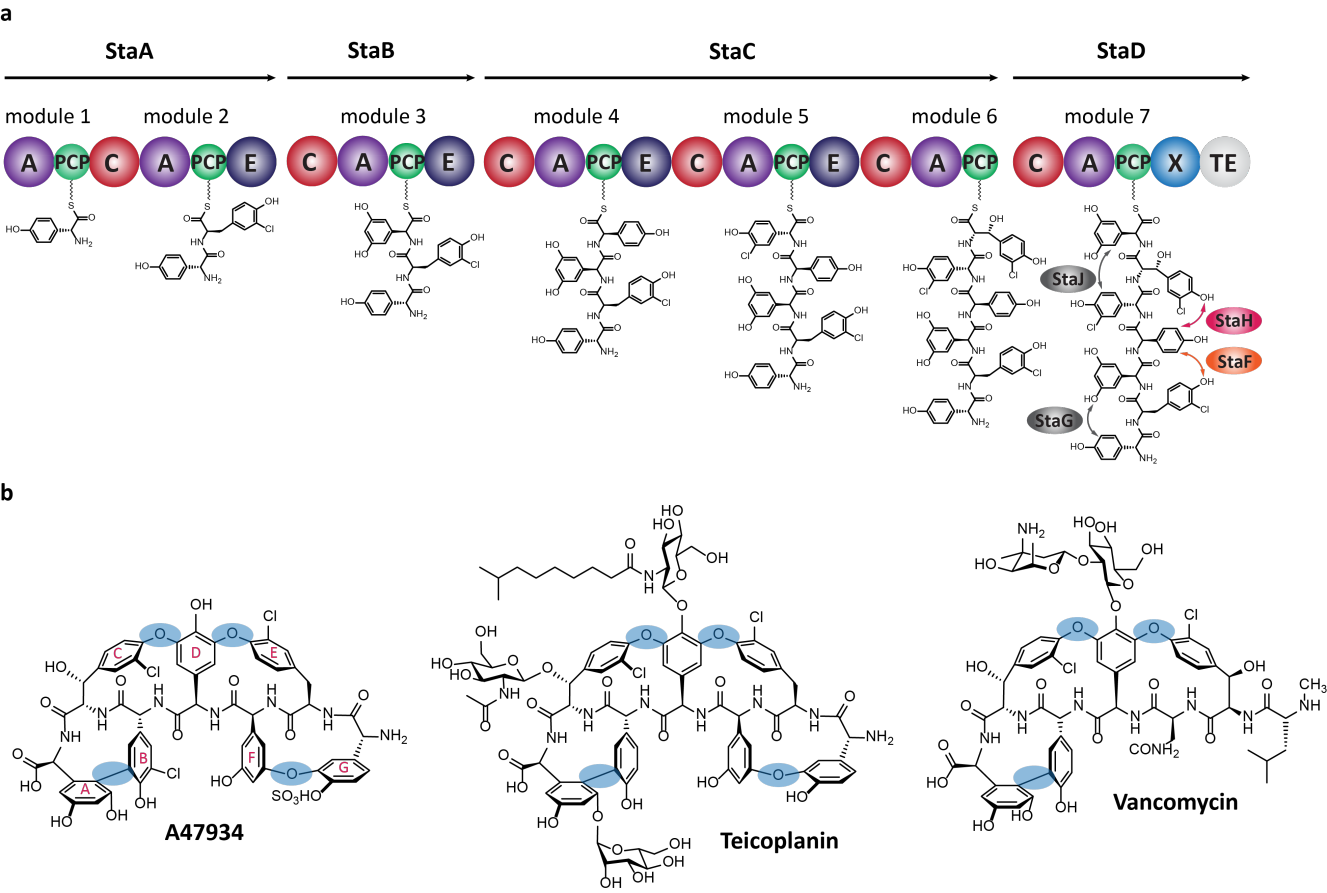
(a) Schematic representation of the biosynthesis of A47934 by the heterotetrameric non-ribosomal peptide synthetase; the 7 modules of the A47934 NRPS machinery are distributed over 4 proteins (StaA to StaD) and exhibit the typical NRPS domain architecture with adenylation (A; purple), peptidyl carrier protein with phosphopantetheine linker (PCP; green), condensation (C; red), epimerisation (E; dark blue), P450-recruitment (X; blue) and thioesterase (TE; light grey) domain; the peptide is shown at its distinct stages of biosynthesis; the amino acid cyclisation steps are depicted with arrows and the corresponding Oxy enzyme; (b) the structures of the glycopeptide antibiotics A47934, teicoplanin and vancomycin, with cross-links highlighted (blue) and standard ring nomenclature shown for A47934 (magenta).

The glycopeptide antibiotics (GPAs) are a series of highly modified heptapeptide natural products and are highly effective antibiotics against Gram-positive bacteria, where they affect their function by preventing the correct crosslinking of the peptidoglycan cell wall [1]. Produced by bacteria, these compounds derive their efficacy from their unique three-dimensional structure, which in turn enables them to bind to the dipeptide terminus of the peptidoglycan precursor lipid II [1–2]. This three-dimensional structure is generated by the high degree of crosslinking exhibited by the glycopeptide antibiotics: in the case of the two most widely known natural examples (vancomycin and teicoplanin) this includes three and four crosslinks, respectively, which occur between the side chains of aromatic residues [3] within the parent heptapeptide (Figure 1b) [4]. This degree of crosslinking in turn renders the total synthesis of GPAs as unfeasible for production and hence both first and second generation GPAs in clinical use are all entirely derived from in vivo biosynthesis [1–2].

The biosynthesis of GPAs is based around the initial synthesis of the linear heptapeptide by a type-I non-ribosomal peptide synthetase (NRPS) [5–6] and its subsequent modification by cytochrome P450 monooxygenases [7–9], which install the crosslinks that provide the unique structure and hence activity of the GPAs (Figure 1a) [4]. Later diversification of the completely crosslinked peptide aglycones is the major source of diversity in natural GPAs, and occurs against the completed peptide aglycones [1,10–11]. The installation of the crosslinks has received significant attention using both in vitro [12–26] and in vivo [27–32] techniques, largely due to the synthetic challenge that these modifications represent. In vivo studies initially confirmed that the cytochrome P450s, known as the Oxy enzymes, are each responsible for the installation of a single ring in the GPA aglycones and that there is a conserved order of activity in both type-I and type-IV GPAs. In type-I GPA biosynthesis OxyB acts first to install the C-*O*-D ring (between residues 4/6), followed by D-*O*-E ring installation (between residues 2/4) catalysed by OxyA and finally formation of the AB ring (between residues 5/7), catalysed by OxyC [28,30–32]. In type-IV systems, where there is an extra ring present between residues 1 and 3 (the F-*O*-G ring), this is installed by OxyE, which acts between OxyB and OxyA in the cyclisation cascade [27]. In vivo experiments also hinted towards the activity of the Oxy enzymes against the substrate peptides whilst they remain bound to the NRPS [29], and in vitro experiments performed with OxyB from the vancomycin biosynthesis pathway confirmed that the Oxy enzymes do indeed act against peptides when these are bound to peptidyl carrier protein (PCP) domains [26]. More recently, it has been shown that the activity of the Oxy enzymes is actually reliant upon an additional conserved domain present within the final module of GPA NRPS machineries, known as the X-domain [16]. Characterisation of this domain has shown that it is a modified, catalytically inactive condensation-type domain and that this domain is capable of forming 1:1 complexes with the Oxy enzymes from GPA biosynthesis [16]. More importantly, with the exception of OxyB_van_, the activity of Oxy enzymes in vitro has also been shown to be highly dependent on the presence of the X-domain fused to the peptidyl carrier protein domain [16]. This in turn has, for the first time, allowed the characterisation of the second cyclisation step, catalysed by OxyA, from the teicoplanin system [13,16–17]. These results showed that OxyA, in contrast to OxyB, is highly selective for the correct stereochemistry of the peptide C-terminal residue and generally displays a higher selectivity for the structure of the substrate peptides [13,17].

Recent in vitro studies performed with the teicoplanin-related A47934 (sta) GPA biosynthetic machinery from *Streptomyces toyocaensis* [33] (Figure 1a) have revealed that the X-domain is in fact far from an innocent bystander during peptide oxidation and that switching this domain to other homologues can affect the selectivity of the Oxy enzymes for their peptide substrates [12]. Combined with the fact that only a single OxyA enzyme has been successfully characterised to date [13–14,16–17], we resolved to make a detailed structural and biochemical analysis of the OxyA homologue from the A47934 system, named StaF, to investigate not only some of the mechanistic features of the OxyA reaction but also the role of the X-domain on the activity of this enzyme and to determine whether the recruitment domain can also affect peptide selectivity for later Oxy enzymes in the GPA cyclisation cascade.

## Results and Discussion

### Spectral analysis of StaF

**Figure 2:**
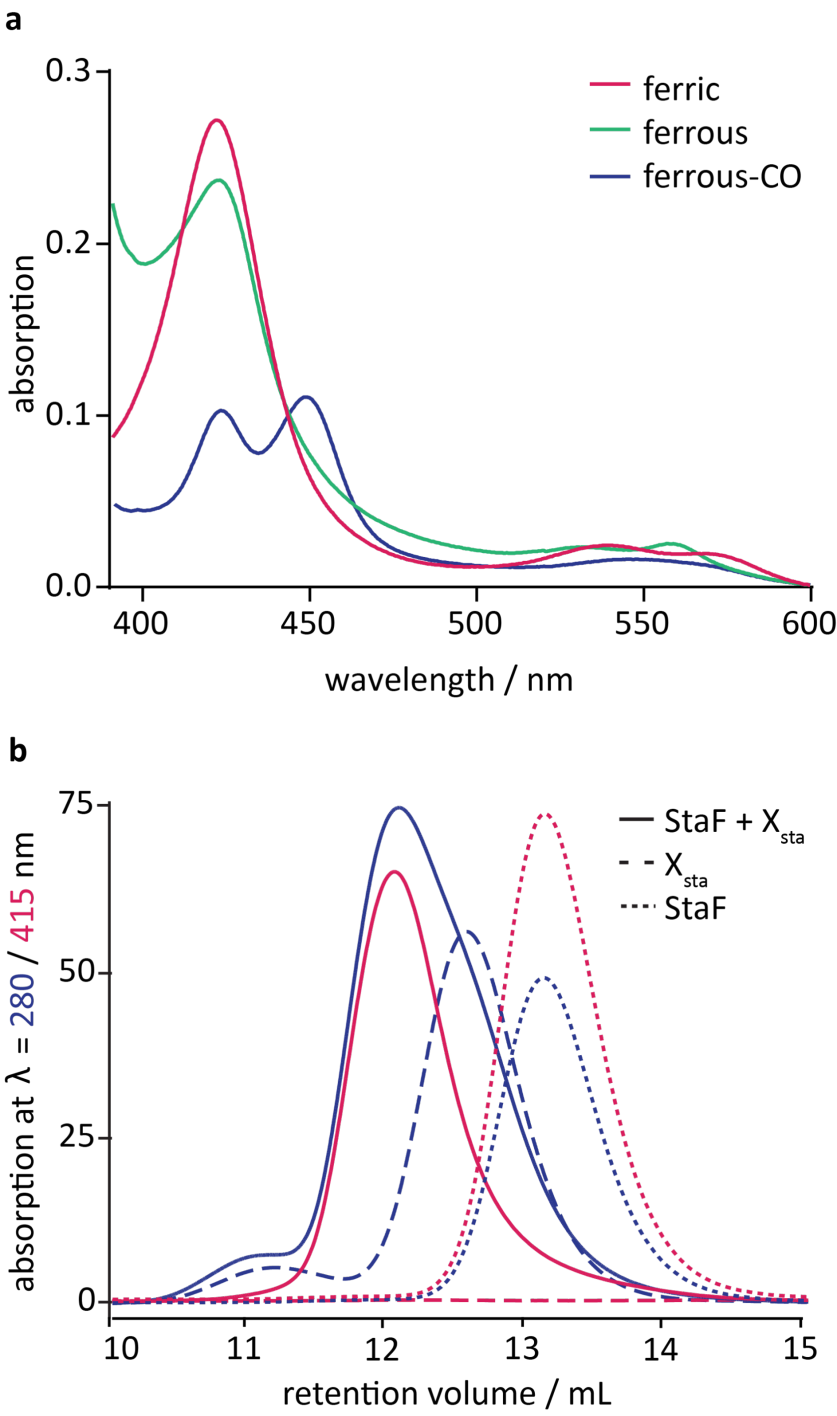
(a) Spectral analysis of StaF, showing the absorption spectra of ferric protein (red), ferrous protein (green) that has been reduced using Na_2_S_2_O_4_, and ferrous protein saturated with CO (ferrous-CO; blue) was measured from λ = 390 to 600 nm; (b) interaction analysis of StaF with the A47934 X-domain; analysis of StaF with a 3-fold excess of X_sta_ was investigated by analytical size-exclusion chromatography measuring absorption at λ = 280 nm (blue) and 415 nm (red; heme-specific); analysis of the individual proteins served as control.

Spectral analysis of P450s allows determination of their potential catalytic competence. The UV–visible spectrum of StaF exhibited a Soret maximum at λ = 421 nm and β/α bands at λ = 539 and 566 nm, respectively (Figure 2a). This corresponds to the absorption spectra characteristic for P450s in the low-spin state, indicating the heme moiety of StaF to be present in its water-bound ferric form. Equivalent spectra were observed for related P450s such as StaH, OxyA_tei_, OxyB_tei_ and OxyE_tei_, which are also involved in GPA cyclisation reactions [12,14,19,22]. Reduction of StaF by addition of sodium dithionite led to conversion of ferric to ferrous heme, which was accompanied by shift of the Soret maximum to λ = 422 nm and of the β/α bands to λ = 532 and 559 nm in the UV–visible spectrum. Upon saturation of ferrous StaF with carbon monoxide, two major peaks appeared at λ = 420 and 450 nm, respectively, as well as a broad minor peak at λ = 548 nm (Figure 2a). The peaks at λ = 420 and 450 nm are caused by different protonation states of the thiol side chain of the proximal heme ligand cysteine: P450 enzymes displaying a protonated thiol ligand (as indicated by a peak at λ = 420 nm) are catalytically inactive, whilst a catalytically competent P450 enzyme with a thiolate-ligated heme exhibits the signature λ = 450 nm absorption peak [34]. The fact that peaks at both λ = 420 and 450 nm appear in the spectrum of StaF indicates that this P450 is present in both incompetent as well as competent states. It has been shown that the inactive form can convert into an active species upon substrate binding [35], however the true catalytic competence of StaF was subsequently determined by substrate turnover assays.

### Interaction analysis of StaF with the A47934 X-domain

StaF, as a member of the group of P450s involved in GPA cyclisation, is anticipated to be recruited to the NRPS machinery through interaction with the X-domain being present in the final NRPS module as has been demonstrated for other Oxy homologues [12–13,16]. In order to determine if StaF is also recruited by the A47934 X-domain (X_sta_) we analysed their interaction by analytical SEC. This method is suitable for interaction analysis with P450s as interaction partner, as not only the typical protein absorption at λ = 280 nm, but also the heme-specific absorption at λ = 415 nm can be monitored. The X_sta_ construct has previously been shown to form a tight interaction with StaH, the P450 responsible for the first (C-*O*-D) phenolic coupling reaction in A47934 biosynthesis [12]. Prior to analysis by SEC, a mixture of StaF and a 3-fold excess of X_sta_ as well as each individual protein was incubated in appropriate buffer (50 mM Tris pH 7.4 and 150 mM NaCl) at room temperature for 30 min to allow complex formation to occur between StaF and X_sta_. Analysis of StaF alone (MW of 47.3 kDa) resulted in overlapping peaks with absorption at λ = 280 and 415 nm at an elution volume of 13.1 mL, whereas when X_sta_ alone (MW of 53.2 kDa) was analysed a λ = 280 nm peak at an elution volume of 12.6 mL was observed. The mixture of StaF with X_sta_ led to the appearance of overlapping peaks with absorption at both λ = 280 and 415 nm at an earlier elution volume of 12.1 mL (Figure 2b), which indicates that the heme-specific λ = 415 nm absorption peak has shifted to an earlier elution volume and that can be explained through formation of a complex between StaF and X_sta_. Thus, we conclude that StaF is, in addition to StaH [12], also recruited to the NRPS machinery through interaction with X_sta_ [16]. The fact that the StaF and X_sta_ mixture shows a single peak upon gel filtration analysis argues for the StaF and X_sta_ molecules being in constant exchange. This is significantly different to the interaction behaviour of StaH and X_sta_, where the interaction of StaH to X_sta_ was strong enough to result in two X_sta_ populations, one bound to StaH and the other free in solution [12]. Studies on the biosynthesis of teicoplanin and the vancomycin-type chloroeremomycin GPA showed decreasing affinity of the P450s to the X-domain with later positions in the GPA cyclisation cascade [13,16], and our results from the A47934 system would appear to follow these trends.

#### Reconstitution of in vitro StaF activity

**Figure 3:**
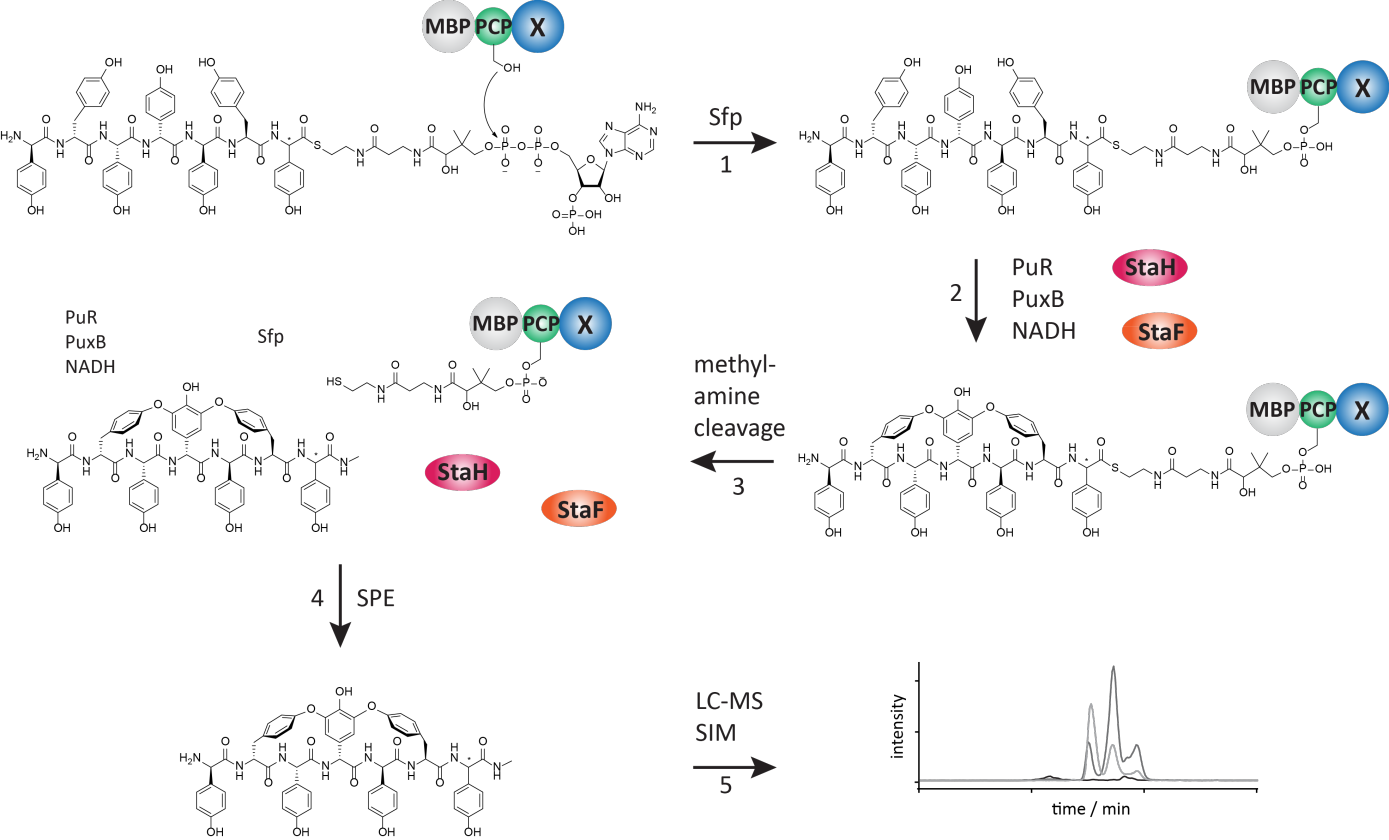
Complete workflow for the Cytochrome P450 activity assay used in this study. 1) Loading of the substrate (Tei7-L/D-Hpg_7_ is depicted) onto a conserved serine residue of the PCP-domain using the R4-4 mutant of the promiscuous phosphopantetheinyl transferase Sfp. The substrate peptide is attached to the PCP-domain via a coenzyme A-derived phosphopantetheine moiety. Excess of substrate is removed via centrifugation using centrifugal filter units with an appropriate MWCO. 2) Subsequently, the activity assay is performed using StaH and StaF together with the redox system composed of palustrisredoxin reductase (PuR), palustrisredoxin B A105V (PuxB) and NADH, in which StaH catalyses C-*O*-D ring formation between D-Hpg_4_ and L-Tyr_6_ and StaF catalyses ring D-*O*-E ring formation between D-Tyr_2_ and D-Hpg_4_. 3) The reaction is quenched by the addition of methylamine, which cleaves off the peptide from the phosphopantetheine linker thus liberating the peptide methylamide. 4) The peptide is purified by solid phase extraction (SPE) and 5) analysed by LC–MS in single ion monitoring (SIM) mode.

**Figure 4:**
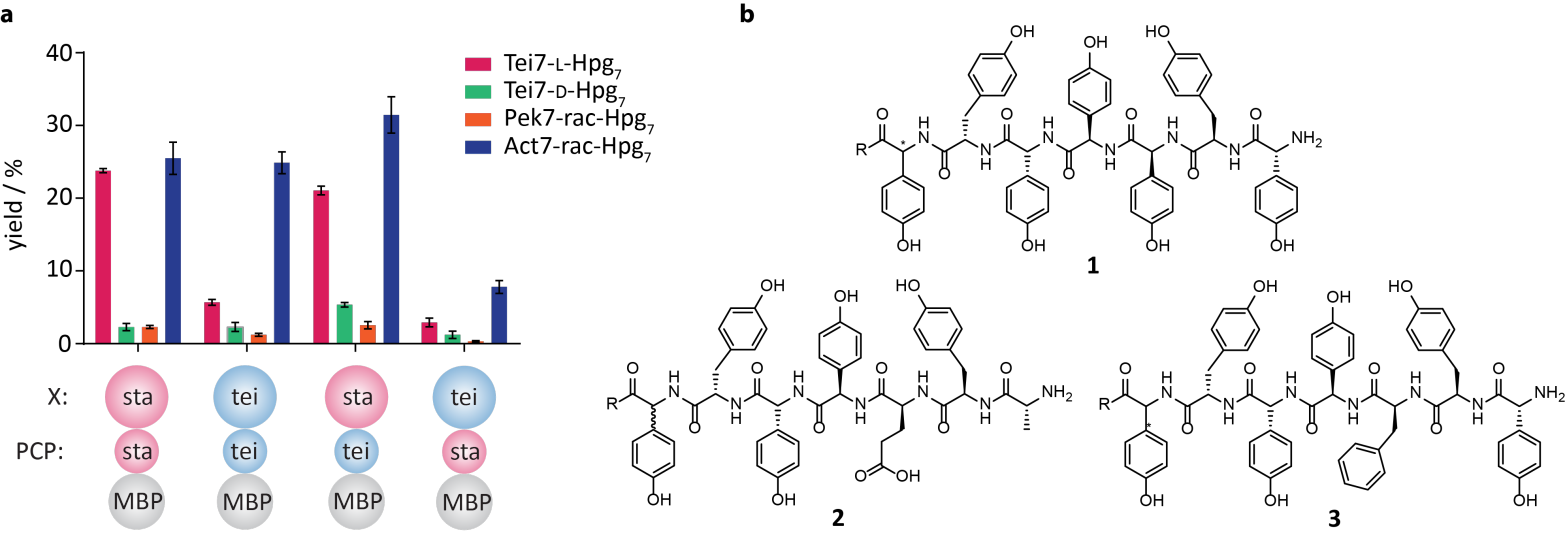
(a) StaF activity against different peptide substrates and using NRPS constructs; the activity of StaF with Tei7-L-Hpg_7_ (magenta), Tei7-D-Hpg_7_ (green), Pek7-rac-Hpg_7_ (orange) and Act7-rac-Hpg_7_ (blue) were determined; all peptides were bound to wildtype and hybrid PCP-X constructs derived from the A47934 (magenta) and teicoplanin NRPS (blue); yield was calculated based on the integrated peak area of bicyclic peptide divided by the sum of the integrated peak areas of monocyclic and bicyclic peptide observed by HPLC–MS (SIM) and is depicted in %; the calculation is based on turnover assay triplicates and the standard deviation is shown. (b) Structures of the peptides used as substrates for StaF, being Tei7-L/D-Hpg_7_ (**1**), Pek7-rac-Hpg_7_ (**2**) and Act7-rac-Hpg_7_ (**3**); R = CoA or methylamine.

On the basis of StaF being a catalytic competent P450 and its interaction with X_sta_, we attempted to reconstitute the activity of this enzyme (Figure 3). In the activity assay we initially employed a teicoplanin-like heptapeptide exhibiting L-Hpg (hydroxyphenylglycine) instead of L-Dpg (3,5-dihydroxyphenylglycine) at position 3 and 7 (abbreviated as Tei7-L-Hpg_7_; Figure 4), which served as suitable substrate as A47934 and teicoplanin exhibit the same amino acid composition of their parent peptide [13,15–18,36]. The linear Tei7-L-Hpg_7_ peptide as well as the mono- and bicyclic products based on P450-catalysed turnover have been analysed in earlier studies [13,16–17]. Prior to the activity assay the substrate was loaded onto the A47934 PCP-X di-domain construct exhibiting maltose binding protein as N-terminal fusion partner (MBP-PCP-X_sta_) using the R4-4 mutant of the promiscuous phosphopantetheinyl transferase Sfp [37]. Subsequently, triplicate turnover assays of StaF both including and excluding StaH were performed using the redox system composed of palustrisredoxin B A105V/palustrisredoxin reductase/NADH to ensure electron supply to the P450s (Figure 3) [38]. NADH was additionally regenerated throughout the assay via a glucose/glucose oxidase couple. The assay was stopped by cleaving the peptide from the PCP-X constructs using excess of methylamine and the peptide was then purified by solid phase extraction before being subjected to HPLC–MS analysis [15,17].

The StaF activity was first investigated using a linear Tei7-L-Hpg_7_ peptide loaded onto the A47934 PCP-X di-domain construct both in the absence and presence of StaH. Only linear peptide was detected in the samples lacking StaH, which is in line with previous in vitro and in vivo experiments that indicate that the presence of the C-*O*-D ring is a prerequisite for the activity of subsequent P450 enzymes, such as StaF (Figure 4, Table 1 entry 1) [13,16,22,27–29,32]. Both mono- and bicyclic peptide products could be detected in samples with StaH included in the turnover assay: given that we have demonstrated that StaH is capable of producing a C-*O*-D ring containing peptide from a linear precursor [12] and the lack of StaF activity against linear peptide substrates, we conclude that the formation of the bicyclic peptide is due to the activity of both StaH and StaF (first by StaH installing the C-*O*-D ring and then subsequent formation of the D-*O*-E ring by StaF, Figure 4). The level of activity observed for StaF was lower than for OxyA_tei_, which is most likely explained by the fact that a significant proportion of StaF was isolated with a protonated – and hence catalytically inactive – heme thiolate ligand. Furthermore, it is also possible that product inhibition could also playing a role in reducing substrate turnover in this system.

**Table 1:** StaF turnover activity.

Entry	fus-PCP-X^a^	peptide	StaF activity^b^

1	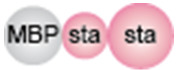	Tei7-L-Hpg_7_	23.8% ± 0.3%
2	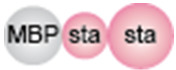	Tei7-D-Hpg_7_	2.3% ± 0.5%
3	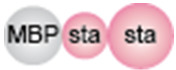	Pek7-rac-Hpg_7_	2.3% ± 0.2%
4	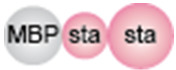	Act7-rac-Hpg_7_	25.5% ± 2.2%
5	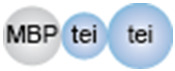	Tei7-L-Hpg_7_	5.7% ± 0.4%
6	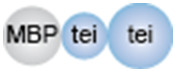	Tei7-D-Hpg_7_	2.3% ± 0.6%
7	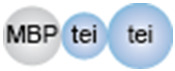	Pek7-rac-Hpg_7_	1.2% ± 0.2%
8	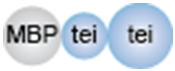	Act7-rac-Hpg_7_	24.9% ± 1.5%
9	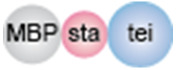	Tei7-L-Hpg_7_	2.9% ± 0.5%
10	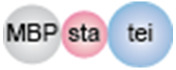	Tei7-D-Hpg_7_	1.2% ± 0.5%
11	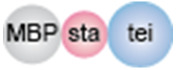	Pek7-rac-Hpg_7_	0.3% ± 0.1%
12	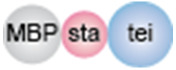	Act7-rac-Hpg_7_	7.8% ± 0.9%
13	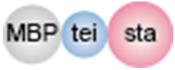	Tei7-L-Hpg_7_	21.1% ± 0.6%
14	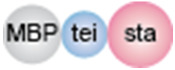	Tei7-D-Hpg_7_	5.4% ± 0.3%
15	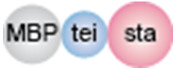	Pek7-rac-Hpg_7_	2.6% ± 0.5%
16	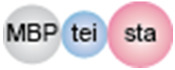	Act7-rac-Hpg_7_	31.5% ± 2.5%

^a^Spheres correspond to the N-terminal fusion partner (abbreviated as *fus*; MBP; shown in grey), PCP- (middle sphere) and X-domain (C-terminal sphere). PCP-/X-domains from A47934 NRPS are shown in red, PCP-/X-domains from teicoplanin NRPS are shown in blue. ^b^Effective StaF activity = integrated peak areas of bicyclic product/sum of integrated peak areas of mono- and bicyclic product observed by HPLC–MS (single ion monitoring). Mean activity and standard deviation were calculated based on turnover assay triplicates (shown in %).

#### Characterising the substrate specificity of StaF

After showing that StaF installs the D-*O*-E crosslink between amino acids D-Tyr_2_ and D-Hpg_4_ on the Tei7-L-Hpg_7_ peptide, we were interested in probing the substrate specificity of StaF and hence we analysed StaF activity on different substrates bound to MBP-PCP-X_sta_ (Figure 4, Table 1, entries 2–4). We found that StaF activity was dramatically reduced with a teicoplanin-like heptapeptide exhibiting the 7^th^ amino acid in the unnatural D-configuration (Tei7-D-Hpg_7_, Figure 4). This indicates that the incorrect stereochemistry of the C-terminal amino acid residue hinders cyclisation of amino acids 2 and 4 in spite of these being localised towards the N-terminus of the peptide, and mimics the behaviour observed for the only other OxyA homologue characterised to date, OxyA_tei_ [13]. This behaviour is in contrast to that of StaH and other OxyB homologues, which exhibit similar activity on both Tei7-L-Hpg_7_ and Tei7-D-Hpg_7_ peptides [12–13,16–17]; StaH even shows a preference for the incorrect peptide diastereomer under specific conditions [12]. These results provide hints of more stringent substrate specificity at later stages of the GPA cyclisation cascade and we hence investigated StaF activity against altered peptide substrates, including pekiskomycin- (Pek) and actinoidin-like (Act) heptapeptides. These peptides differ to A47934 and teicoplanin in the amino acid residues present in positions 1 and 3 of the peptide (Pek: 1, 3; Act: 3; Figure 4) [1,17,39]. Both Pek- and Act-heptapeptides exhibit a Hpg residue at position 7 instead of L-Dpg, but with a racemic mixture of L- and D-Hpg_7_ (Pek7-rac-Hpg_7_, Act7-rac-Hpg_7_) due to an inability to resolve the diastereomers via HPLC. We observed very little activity of StaF against Pek7-rac-Hpg_7_ (Figure 4, Table 1, entry 3), which is similar to the results observed for OxyA_tei_. This could be explained by the significant differences in the structures of the amino acids at positions 1 and 3 of the peptide, given that these are in the direct locale of the residues involved in the D-*O*-E ring [17]. In contrast, StaF (following StaH-catalysed monocyclisation of the linear peptide) showed similar activity against Act7-rac-Hpg_7_ (Figure 4, Table 1, entry 4) compared to Tei7-L-Hpg_7_, which indicates a preference for hydrophobic amino acids with bulky side chains. This is clearly different to the broad substrate specificity shown by the preceding enzyme StaH, which accepts peptides including Tei7-L/D-Hpg_7_, Pek7-rac-Hpg_7_ as well as Act7-rac-Hpg_7_ as shown here [12]. Similar results were obtained for OxyB and OxyA from the teicoplanin system and it appears that the substrate specificity of the P450s is decreased when acting on later steps of the GPA cyclisation cascade [17]. This also makes the identification of an active OxyA homologue from a type-I GPA producer (vancomycin/pekiskomycin type) [1] of great importance to test the selectivity of these homologues against altered peptide substrates.

#### Impact of the A47934 X-domain on StaF activity

Previously, it has been shown that StaH exhibits high activity against peptide substrates presented by the PCP-X-di-domain from teicoplanin biosynthesis, whilst low activity was achieved on PCP-X constructs from the A47934 biosynthetic machinery. Through domain exchange of PCP-X constructs from the A47934 and teicoplanin NRPS system, it was discovered that the A47934 X-domain was responsible for the low levels of StaH activity [12]. In order to analyse if this effect is maintained over the subsequent amino acid cyclisation reactions in A47934 biosynthesis, we tested StaF activity using the same constructs all exhibiting MBP as N-terminal fusion partner: a PCP-X construct from A47934 biosynthesis (MBP-PCP-X_sta_, Table 1, entries 1–4), a PCP-X construct from teicoplanin biosynthesis (MBP-PCP-X_tei_, Table 1, entries 5–8) and hybrid PCP-X constructs from A47934 and teicoplanin biosynthesis (MBP-PCP_sta_-X_tei_, Table 1, entries 9–12; MBP-PCP_tei_-X_sta_, Table 1, entries 13–16;) [12]. The influence of each individual PCP-X construct was tested with Tei7-L/D-Hpg_7_, Pek7-rac-Hpg_7_ and Act7-rac-Hpg_7_ peptides. The presentation of Tei7-D-Hpg_7_ and Pek7-rac-Hpg_7_ by MBP-PCP-X_tei_, MBP-PCP_sta_-X_tei_ and MBP-PCP_tei_-X_sta_ did not lead to a change in their acceptance by StaF, with both peptides not accepted as substrates (Figure 4, Table 1, entries 6, 7, 10, 11, 14, and 15). In case of Tei7-L-Hpg_7_, StaF was active when the substrate was bound to MBP-PCP-X_sta_, as described above, and we also observed a similar activity level when the peptide was presented by the MBP-PCP_tei_-X_sta_ construct (Table 1, entry 13). However, against our expectations, StaF activity did not increase on Tei7-L-Hpg_7_ when bound to PCP-X constructs exhibiting the teicoplanin X-domain (MBP-PCP-X_tei_/PCP_sta_-X_tei_), but rather showed a significant decrease (Figure 4, Table 1, entries 5 and 9): StaF activity is clearly diminished when using the non-matched X-domain. In order to determine if this effect was maintained with different peptide substrates, we analysed StaF activity using the Act7-rac-Hpg_7_ peptide: StaF activity was highest with MBP-PCP_tei_-X_sta_, showed a minor decline with MBP-PCP-X_sta_ and MBP-PCP-X_tei_ and was considerably decreased with MBP-PCP_sta_-X_tei_ (Figure 4, Table 1, entries 16, 4, 8 and 12, respectively). Thus, the negative effect of the teicoplanin X-domain on StaF activity with Act7-rac-Hpg_7_ is not as clear as for Tei7-L-Hpg_7_. An explanation for this could lie in the observation that Act7-rac-Hpg_7_ seems to be a very good substrate for StaF (as it is for OxyA_tei_), possibly due to the increased conformational flexibility of the phenylalanine residue at position 3 of the peptide when compared to the Hpg residue present in the teicoplanin-like peptide (Figure 4). In spite of this, Tei7-L-Hpg_7_ is the peptide with the highest structural similarity to the natural substrate and the fact that StaF activity on Tei7-L-Hpg_7_ was obtained only with PCP-X constructs exhibiting the A47934 X-domain indicates that StaF is dependent on the corresponding X-domain from its own NRPS when using teicoplanin-like peptides. Comparison of StaH and StaF activity reveals that while StaH exhibits only low to moderate activity on substrates bound to PCP-X constructs with the A47934 X-domain, presence of the A47934 X-domain in PCP-X constructs appears to be essential for StaF activity. In case of StaH, the high affinity of the A47934 X-domain likely hinders reorganisation of the P450/NRPS complex and hence can be trapped in states that display sub-optimal substrate orientation in the P450 active site [12]. In the case of StaF, it now seems clear that the natural X-domain is in fact the best system for peptide cyclisation, although different combinations of X-domain and peptide substrate can be identified that afford atypical levels of in vitro activity (StaF: Act7-rac-Hpg_7_ peptide and A47934 X-domain; StaH: Tei7-D-Hpg_7_ peptide and A47934 X-domain). Thus, our findings highlight the importance of the X-domain in GPA cyclisation reactions and provide further indication that its role appears to be more than just recruitment of the P450 to the substrate, but also ensuring proper substrate orientation via the PCP-domain in the P450 active site.

#### Structure and active site architecture of StaF

In order to gain insight into the structure-function relationship of StaF, we attempted to structurally characterise the protein. We were able to determine the crystal structure of StaF to a resolution of 2.1 Å and 2.2 Å using different cryo-protectant solutions (ethylene glycol and glycerol) and by solving the phase problem through molecular replacement using OxyE_tei_ (PDB ID: 3O1A) as search model (Table 2) [22]. Both structures exhibit a core RMSD of only 0.2 Å, indicating that the structures are practically identical. Manual comparison also did not reveal any important differences between them and thus we used the highest resolution structure for analysis (PDB ID: 5EX8; ethylene glycol cryo-protectant solution).

**Table 2:** Crystallographic data for StaF.

**Data collection**	StaF Native ethylene glycol	StaF Native glycerol

Space group	*P*3_1_2_1_ (152)	*P*3_1_2_1_
Cell dimensions *a, b, c* (Å)	110.1, 110.1, 93.7	109.7, 109.7, 93.9
Molecules/asymmetric unit	1	1
X-ray source	SLS X10SA	SLS X10SA
Wavelength (Å)	0.9792	0.9792
Resolution (Å)^a^	50.0–2.1	50.0–2.2
*R_merge_*^a^	0.07 (0.31)	0.10 (0.44)
*I/σI*^a^	19.8 (4.2)	16.8 (5.5)
Completeness (%)^a^	95.1 (91.6)	98.4 (95.5)
Redundancy	6.2	9.6
Wilson *B*-factor (Å^2^)	27.6	35.3
**Refinement**		

Unique Reflections	36206	31857
Resolution in refinement	50.0–2.1	50.0–2.2
*R_work_*/*R_free_*^b^(%)	19.4 / 22.9	19.1 / 22.0
TLS-groups	−22–18; 19–75; 79–152; 153–225; 226–326; 327–391	−22–25; 26–75; 79–107; 108–206; 207–320; 321–391
**No. of atoms**	3690	3576
Protein Heme Ethylene glycol Glycerol Water	3315 43 64 – 268	3269 43 – 48 216
***B-*factors**		
Protein Heme Ethylene glycol Glycerol Water	35.1 21.5 53.0 – 43.8	35.5 22.0 – 59.8 41.7
**RMSD**		
Bond lengths (Å) Bond angles (°) Ramachandran statistics^c^ Ramachandran statistics^d^	0.009 1.193 97.3/ 2.2/ 0.5^e^ 97.1/ 2 / 3	0.008 1.107 97.8/ 1.7/ 0.5^f^ 97.6/ 2 / 6
PDB Code	5EX8	5EX9

^a^Numbers in parentheses correspond to the highest resolution shell (2.2–2.1 Å; 2.3–2.2 Å). ^b^*R_work_* = ∑ ||F_o_|-|F_c_||/ ∑|F_o_|, calculated from the working reflection set; *R_free_* calculated in the same manner using the 5% test set reflections. ^c^Calculated by PROCHECK; percentage of the protein residues in favored/ allowed/ disallowed regions. ^d^Calculated by MOLPROBITY; percentage of the protein residues in most favored regions; disallowed residues and percentage of bad rotamers. ^e^Residues in disallowed region: E331 (disordered loop region), F382 (active site residue, clearly defined density). ^f^Residues in disallowed region: A329 (disordered loop region), F382 (active site residue, clearly defined density).

**Figure 5:**
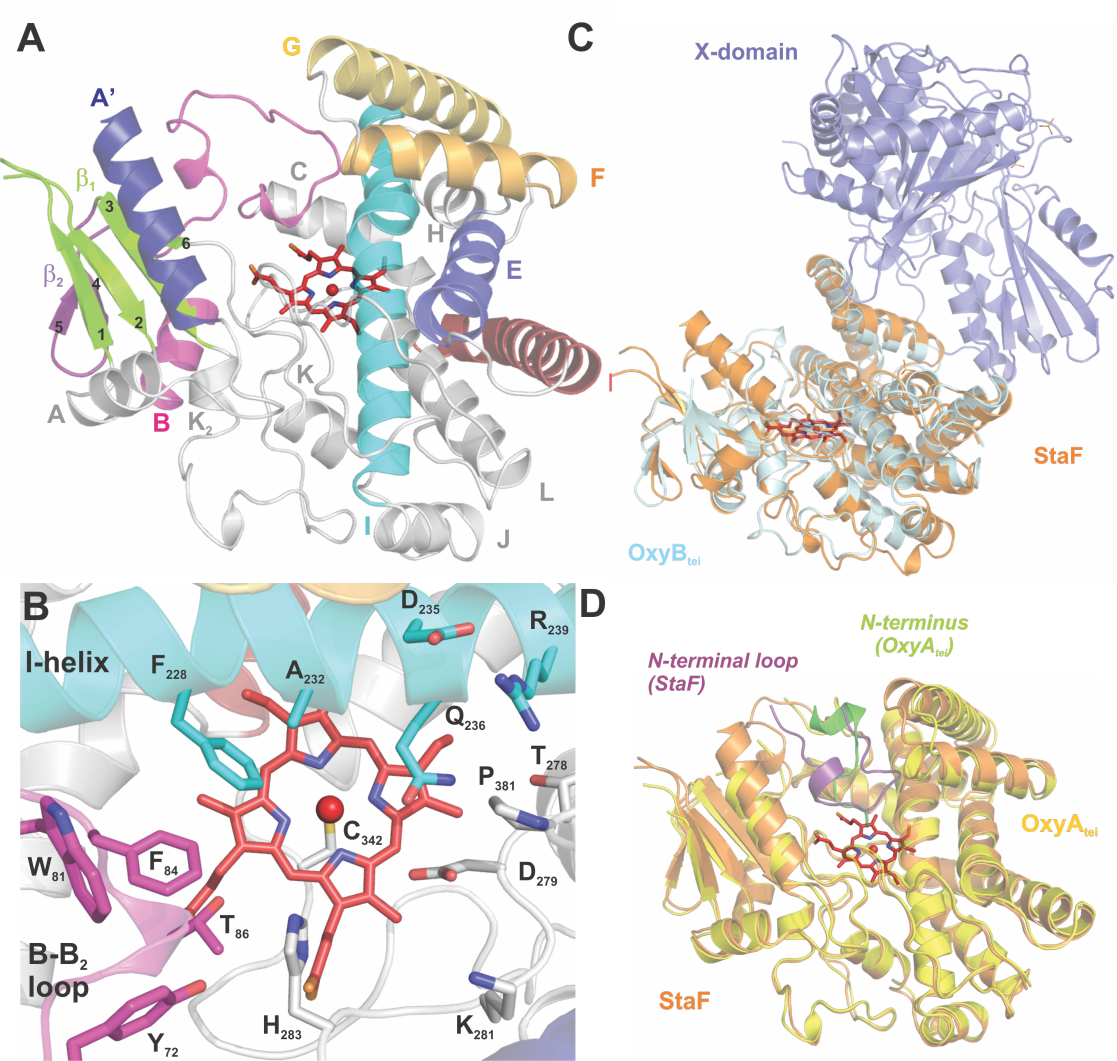
Structural analysis of StaF: (A) overall structure of StaF, with the heme moiety depicted using sticks and specific helices coloured and labelled; (B) view of the active site of StaF, with residues close to the heme moiety shown as sticks and labelled, with the colour scheme and labelling retained from panel (A); (C) an overlay of the StaF structure (orange) on the X_tei_-OxyB_tei_ complex (pale cyan/ blue; PDB ID: 4TX3); (D) an overlay of StaF (orange) on the structure of OxyA_tei_ (yellow) showing the location of the N-terminal regions of other molecules within the crystal lattice for both StaF (purple) and OxyA_tei_ (green).

The StaF structure is well resolved and adopts the typical structure of a cytochrome P450 [34], which consists predominantly of α-helices (12 in total: labelled A to L, including two additional helices labelled A’ and J’, Figure 5A). The core of the P450, the four-helix bundle, is present in StaF and comprises helices D, E, I and L. Two β-sheet regions are observed on the side of the protein opposite to the core 4-helix bundle of the P450, with β-1 exhibiting 4 strands and β-2 exhibiting 2 strands. The most interesting structural feature of StaF is the long A’ helix at the N-terminus, which forms the ceiling of the active site. This helix seems to be specific for D-*O*-E ring catalysing P450s as it was only observed once before in OxyA_tei_, the D-*O*-E ring forming P450 from teicoplanin biosynthesis [14]. The centre of the active site is occupied by a heme moiety, which is sandwiched between helix I and L, the loops connecting helices B and C, K and L as well as the loop connecting the last β-strand of β-1 and the J’ helix. The thiolate side chain of Cys342 serves as proximal ligand for the heme and is found in the conserved P450 heme-binding sequence (FGHGxHxCLG) in the K–L loop. The heme propionate moieties also interact with the protein through ionic interactions: His93 (2.7 Å), Arg97 (2.8 Å), His283 (2.7 Å), Arg285 (2.7 and 2.9 Å) and His340 (2.8 Å).

The architecture of the active site involves the I helix, the B–C loop and the loop connecting the J’ helix and the last strand of β-1. Its ceiling is formed by the A’, F and G helices and the C-terminal loop of the protein (Figure 5B). Phe382, present in the long C-terminal loop that impinges on the active site, adopts an unusual Ramachandran conformation. As this conformation is also found for OxyA_tei_ (PDB ID: 5HH3) and forms a portion of the active site, this is likely to be of importance for the activity of these enzymes. The other region where Ramachandran outlines are present in the structure of StaF (329-331) is in the region prior to the crucial heme-coordinating cysteine residue Cys342, which is a region of poorly defined electron density. The I helix contains the conserved residues responsible for controlling protonation during oxygen activation of the P450 catalytic cycle (Asp235 and Gln236) [34]. Residues projecting into the active site are Thr86 in the B–C loop, Gly231 in the I helix and Asp279 and Thr282 in the loop connecting the J’ helix and β-1. These residues make the active site more polar than those of OxyB/OxyC homologues, whilst aromatic amino acids are concentrated at the B–C-loop side of the active site, with Trp81 and Phe84 in the B–C loop and Phe228 in the I helix. This distribution of polar and hydrophobic residues in the active site is clearly different from the arrangement in related P450s such as StaH (PDB ID: 5EX6), OxyB_tei_ (PDB ID: 4TVF) and OxyB_van_ (PDB ID: 1LG9) [12,19,40–41], where hydrophobic residues were concentrated in the middle of the active site around the heme, and was only previously observed in OxyA_tei_ (PDB ID: 5HH3) [14].

#### Structural comparison to other P450s

The presence of the additional A’ helix and the distinct distribution of polar and aromatic amino acid residues in the active site sets StaF and OxyA_tei_ apart from other structurally characterised examples of P450s involved in GPA cyclisation reactions. Comparison of StaF and OxyA_tei_ (PDB ID: 5HH3) reveals very similar structures with a core rmsd of 1.2 Å. Major differences include the length of the N-terminus, which is shorter for StaF, the conformation of the B–C loop, which exhibits a helical part in OxyA_tei_ in contrast to StaF, and the position of the F and G helices, which are drawn down towards the centre of the protein in StaF closing the active site to a greater extent than observed for OxyA_tei_. In both the StaF structures, the N-terminal (tag) region of a symmetry-related molecule forms a loop above the heme, which likely leads to the open conformation of the B–C loop region (Figure 5D). One of the protein chains in the asymmetric unit of the OxyA_tei_ structure also displays an interaction with the N-terminus of another protein chain, although in this case there is direct coordination between the N-terminal amine nitrogen and the heme iron. This different binding mode leads to minor changes in the orientation of various amino acid side chains within the active site of OxyA_tei_ when compared to StaF as well as the opening of the F-G helices and alterations to the I-helix packing (Figure 5D). An attempt to reengineer the protein construct to shorten the N-terminal protein tag and to redesign the sequence to resemble that of a PCP domain both lead to proteins that failed to crystallise either under the original conditions or in broad screens. Thus, it would appear as though OxyA homologues require active site interactions in order to stabilise their structures sufficiently to enable crystallisation, which is in contrast with other Oxy homologues. The importance of active site interactions may also provide an indication why OxyA enzymes appear to have higher degrees of substrate specificity than OxyB homologues.

**Figure 6:**
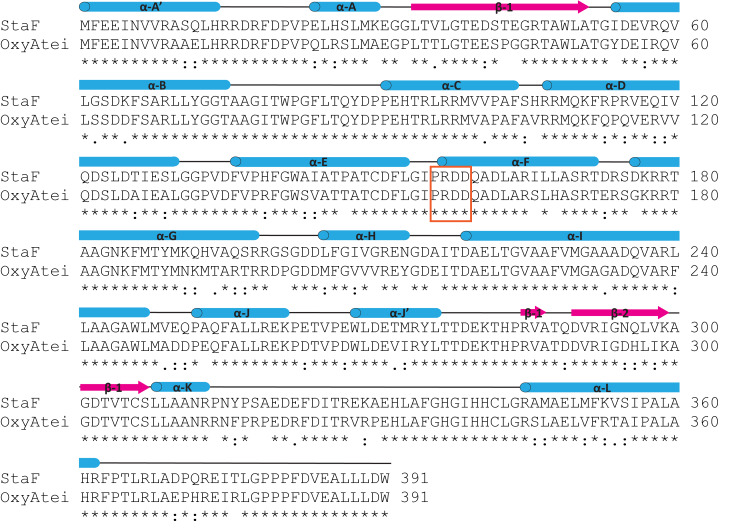
Sequence alignment of StaF and OxyA_tei_. Protein secondary structure was derived from the StaF crystal structure (PDB ID: 5EX8) and is shown above the alignment (α-helices = blue, β-sheets = magenta). The PRDD-region, which has been shown to be crucial for interaction with the X-domain is highlighted in an orange box.

**Figure 7:**
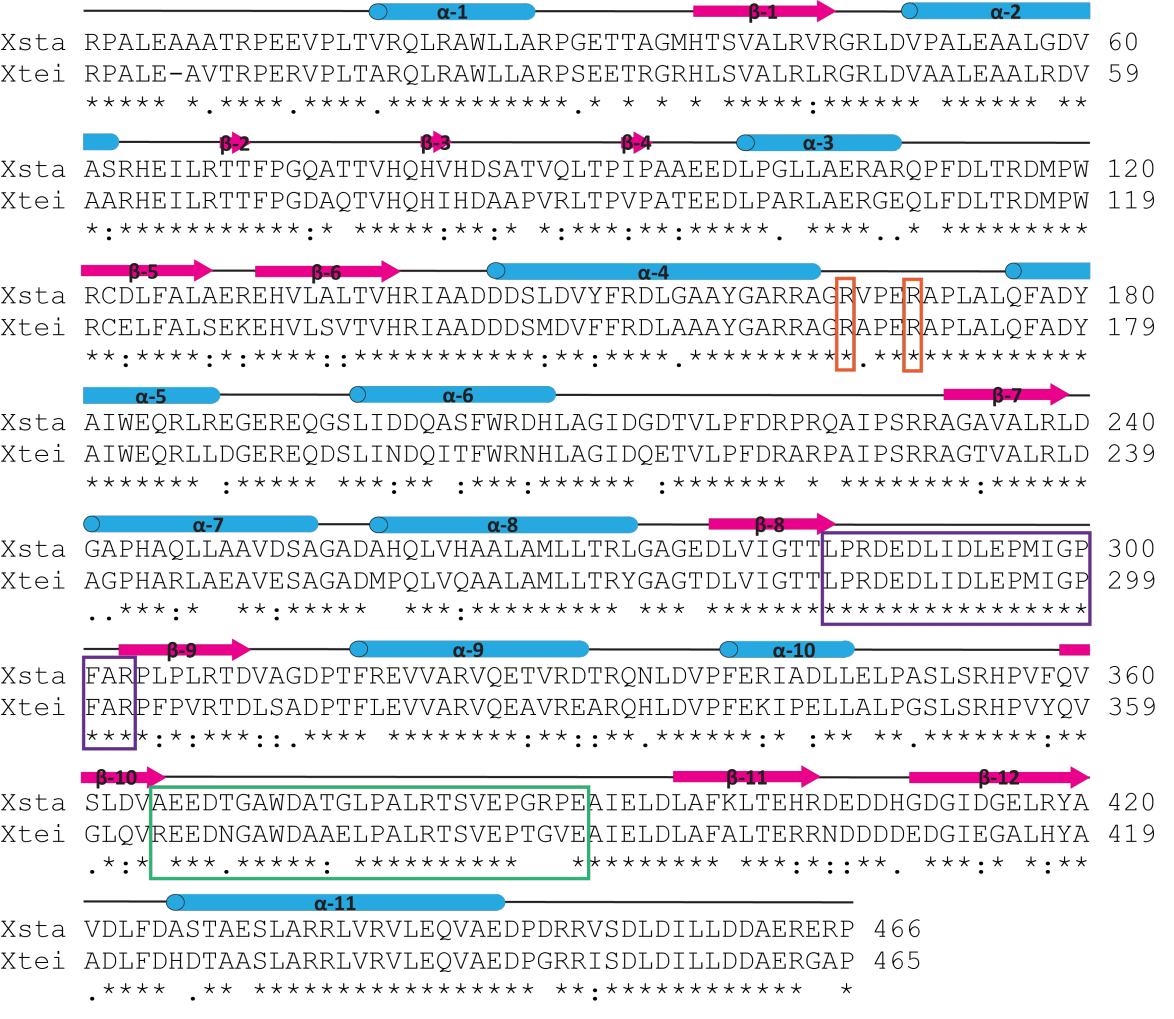
Sequence alignment of the A47934 (sta) and teicoplanin (tei) X-domain; secondary structure was derived from the X_tei_-OxyB_tei_ complex (PDB ID: 4TX3) and is shown above the alignment (α-helices = blue, β-sheets = magenta); the residues crucial for interaction with cytochrome P450s are shown in orange and both the crossover I region (purple) and the crossover II region (green) are highlighted.

The StaF structure is similar to the structures of other Oxy homologues that have been solved [4], including OxyE_tei_ (PDB ID: 3O1A/3OO3) [21–22] and OxyB_tei_ in complex with the X-domain (PDB ID: 4TX3) [16] with an rmsd of under 2.0 Å (Table 3). Other P450 enzymes with high structural similarity to StaF are those from secondary metabolism and involve oxidative functionalisation of large substrates, such as pravastatin (CYP105AS, PDB ID: 4OQS) [42], oleandomycin (OleP, PDB ID: 4XE3) [43], mycinamicin (MycG, PDB ID: 2YCA) [44] and filipin (CYP105P1, PDB ID: 3E5L) [45] (Table 3). StaF also shows moderate levels of structural similarity to other P450s that oxidise carrier protein-bound substrates, including the fattyacyl-ACP oxidase P450_BioI_ (PDB ID: 3EJD) [46–48] and the aminoacyl-PCP hydroxylases OxyD (PDB ID: 3MGX) and P450_sky_ (PDB ID: 4PXH) [49–50] (Table 3). Central to the Oxy/X-domain interaction is the PRDD-region, which is found at the beginning of the F-helix in the Oxy enzymes [16]. This motif is conserved in the Oxy enzymes, and the two Asp residues located in this region form numerous contacts to the X-domain [16]. Overlaying the structure of StaF onto the OxyB_tei_/X-domain complex structure shows that the interface expected between StaF and the teicoplanin X-domain would appear to be a favourable one, although this is clearly not the case based on the data from in vitro activity assays (Figure 5C). Sequence-based comparisons of OxyA_tei_ and StaF (Figure 6) as well as the A47934 and teicoplanin X-domains (Figure 7) also do not provide a clear indication of the grounds of the selectivity of StaF for the A47934 X-domain over that from the teicoplanin system. However, the discovery that peptides can be accepted by StaF when presented by the teicoplanin X-domain if the correct peptide sequence is selected (specifically the Act7-rac-Hpg_7_ peptide) shows that the peptide plays a significant role in the formation of a catalytically competent state of StaF. This cannot be explained by the current structures that we have access to from GPA biosynthesis. This also clearly indicates the importance of characterising substrate-bound Oxy structures in future, although this remains a challenging task.

**Table 3:** Top ranking structures homologous to StaF as identified by a Dali search.

PDB code	Chain	RMSD Cα [Å]	Z-score	% Identity	Description (donor organism)	Ref

5HH3	A	1.2	55.9	79	OxyA_tei_ (*Actinoplanes teichomyceticus*)	[14]
5HH3	C	1.6	55.7	80	OxyA_tei_ (*Actinoplanes teichomyceticus*)	[14]
3OO3	A	1.8	46.3	48	CYP165D3 (*Actinoplanes teichomyceticus*)	[21]
3O1A	A	2.0	46.3	48	CYP165D3 (*Actinoplanes teichomyceticus*)	[22]
4TX3	A	1.9	44.0	41	OxyB_tei_ in complex with the X-domain (*Actinoplanes teichomyceticus*)	[16]
1LG9	A	2.6	43.6	38	CYP165B3 (*Nocardia orientalis*)	[41]
5EX6	A	2.2	43.4	41	StaH (*Streptomyces toyocaensis* NRRL15009)	[12]
1UED	A	2.1	43.2	33	CYP165C3 (*Nocardia orientalis*)	[40]
4OQS	A	2.2	42.1	34	CYP105AS1 *(Amycolatopsis orientalis)*	[42]
4TVF	A	2.1	41.2	42	OxyB_tei_ (*Actinoplanes teichomyceticus*)	[19]
4XE3	B	2.4	41.1	30	OleP (*Streptomyces antibioticus*)	[43]
2YCA	A	2.3	40.8	28	MycG (*Micromonospora griseorubida*)	[44]
3E5L	A	2.5	40.7	34	CYP105P1 (*Streptomyces avermitilis*)	[45]
3EJD^a^	B	2.4	38.5	25	P450_BioI_ (CYP107H1, *Bacillus subtilis*)	[47]
3MGX^a^	B	2.9	35.6	22	OxyD (CYP146, *Amycolatopsis orientalis*)	[51]
4PXH^a^	E	3.0	35.5	19	PCP_7_-P450_sky_ complex (CYP163B3, *Streptomyces sp. ACTA 2897*)	[49]

^a^Included for purposes of comparison.

## Conclusion

In this study we characterised the activity and structure of StaF, the D-*O*-E ring forming Oxy enzyme from A47934 biosynthesis. This is only the second characterised example of these types of P450s, after the teicoplanin homologue OxyA_tei_. StaF adopts the canonical P450 fold and strongly resembles the structure of OxyA_tei_, with both exhibiting the long additional A’ helix at the protein’s N-terminus. Spectral analysis of StaF showed that it exhibits the typical P450 absorption spectra, but with only half of the StaF species being in the catalytically competent state upon reduction and CO-complexation. Despite this, we successfully reconstituted the StaF activity in vitro and could show that the substrate specificity of StaF is not as broad as for Oxys catalysing the C-*O*-D ring formation, in agreement with the results from OxyA_tei_. Additionally, we could show that StaF interacts with the A47934 X-domain, indicating that StaF is, along with other related Oxy enzymes, recruited by the X-domain to the A47934 NRPS machinery. The interaction of StaF to X_sta_ appears to be weaker than the interaction of StaH to X_sta_. We have previously shown that the strong StaH/X_sta_ interaction is the cause for poor substrate turnover of StaH of substrates bound to PCP-X constructs exhibiting the A47934 X-domain. In contrast, the weaker interaction of StaF to X_sta_ helps to explain why StaF exhibits higher levels of activity against substrates bound to PCP-X construct exhibiting the A47934 X-domain. Taking into account the weaker binding of StaF to X_sta_, we postulate that the weaker interaction of this complex allows substrate reorganisation after initial complex formation, which ensures proper substrate orientation in the active site. These results highlight the importance of testing different peptide/protein carrier constructs for in vitro GPA cyclisation assays and show that different Oxy homologues, such as StaH and StaF, can display significantly different reactivity and specificity despite their similar sequences, structures and substrates. Such insights will be crucial in future identification of an optimal system for the in vitro generation of GPAs.

## Experimental

### Cloning

The gene encoding StaF was obtained from genomic DNA [33] and was amplified by PCR using specific primers (fwd: 5’-CACCATGTTCGAGGAGATCAACGTCGTC-3’, rev: 5’- CTACCAGTCGAGCAGCAGGGCTTC- 3’) for cloning into pET151d (Life Technologies) using TOPO-cloning. The plasmid was sequenced using T7 promoter and terminator primers. StaF was expressed with an N-terminal hexahistidine-tag and under the control of the T7 promoter. The StaH construct (pET28a StaH) as well as all NRPS constructs (pET MBP-PCP_sta_-X_sta_ 1c, pET MBP-PCP_tei_-X_tei_ 1c, pET MBP-PCP_tei_-X_sta_ 1c, pET MBP-PCP_sta_-X_tei_ 1c, pET NCL-4 MBP-X_sta_) were employed from a previous study – Ulrich et al. (2016) [12].

### Expression and purification

*StaF.* For the expression of StaF, a starter culture of *E. coli* KRX cells (Promega), which had been transformed with pET151d StaF, was grown at 37 °C overnight. This was used for the inoculation of 6 × 2 L TB medium plus 100 mg/L ampicillin with 1% (v/v) of starter culture. This expression culture was incubated at 37 °C and 90 rpm until an OD_600_ = 0.4 was reached. At this point, 25 mg/L δ-aminolevulinic acid was added and the temperature was decreased to 18 °C. The culture was further grown until an OD_600_ = 0.6–0.8, at which the expression was induced with 0.1% rhamnose and 0.1 mM IPTG. After overnight expression, cells were harvested at 5000*g* and 4 °C for 10 min and resuspended in lysis buffer (50 mM Tris pH 8, 50 mM NaCl, 10 mM imidazole, 0.5 mM DTE, EDTA-free SIGMAFAST™ Protease Inhibitor Cocktail Tablet).

All purification steps were performed at 4 °C if not stated otherwise. First, the cells were lysed by 3 passes through a microfluidizer (Microfluidics, Westwood, USA), before the lysate was centrifuged at 20,000*g* for 30 min. The cleared lysate was then subjected to Ni-NTA affinity chromatography in batch mode to purify the N-terminally hexahistidine-tagged StaF. Therefore, the Protino^®^ Ni-NTA Agarose resin (Macherey-Nagel, Düren, Germany) was equilibrated twice with the 10-fold column bed volume (CV) of Ni-NTA wash buffer (50 mM Tris pH 8, 300 mM NaCl, 10 mM imidazole). This was achieved through resuspension of the resin in the Ni-NTA wash buffer and subsequent removal of the Ni-NTA wash buffer after centrifugation at 1000*g* for 1 min. Subsequently, the Ni-NTA resin was incubated with the cleared lysate for 1 h and rotation. The supernatant was then removed by centrifugation as described above, before the Ni-NTA resin was washed with 10 × CV of Ni-NTA wash buffer for 5 min with rotation. Prior to transfer of the Ni-NTA resin into column format, the Ni-NTA wash buffer was removed by centrifugation as described above and resuspended in 2 × CV Ni-NTA wash buffer. StaF was finally eluted using 3 × CV of Ni-NTA elution buffer (50 mM Tris pH 8, 300 mM NaCl, 300 mM imidazole).

For anion exchange chromatography (AEC), the Ni-NTA elution was buffer exchanged with AEC buffer A (see below) using illustra NAP-25 columns (GE Healthcare, Chalfont St Giles, UK) and concentrated using vivaspin^®^ centrifugal concentrators with a 30 kDa MWCO (Sartorius, Göttingen, Germany). AEC was then performed using a Resource™ Q (6 mL) column (GE Healthcare, Chalfont St Giles, UK) connected to an Äkta pure 25 system with 50 mM Tris pH 7.4, 20 mM NaCl as AEC buffer A and 50 mM Tris pH 7.4, 1 M NaCl as AEC buffer B at rt. The column was equilibrated with AEC buffer A, before the protein solution was applied onto the column. The column was then washed with 5 × CV of AEC buffer A, before StaF was eluted using a gradient of 20 × CV of 0 to 100% AEC buffer B. Appropriate elution fractions were pooled and concentrated as described above, after analysis by SDS-PAGE.

Additionally, StaF was further purified by size-exclusion chromatography (SEC) using a Superose 12 (300 mL) column connected to an Äkta pure 12 system at rt. The column was equilibrated with SEC buffer (50 mM Tris pH 7.4, 150 mM NaCl), before the protein solution was applied onto the column. StaF was then eluted using SEC buffer. The elution fractions were again analysed by SDS-PAGE, appropriate fractions were pooled and concentrated as described above. Determination of the protein concentration was performed spectroscopically using a Nanodrop spectrophotometer (Thermo Fisher Scientific, Waltham, USA) and the calculated extinction coefficient of the protein at λ = 280 nm. Furthermore, the protein identity was confirmed by MALDI–TOF MS peptide map fingerprinting of a tryptic digest of excised protein bands from SDS-PAGE analysis. StaF was finally stored in SEC buffer in aliquots, which were first flash frozen in liquid nitrogen before being stored at −80 °C. The yield of purified StaF was 28 nanomoles (1.3 mg) per L of expression culture.

*StaH, MBP-PCP_sta_-X_sta_, MBP-PCP_tei_-X_tei_, MBP-PCP_tei_-X_sta_, MBP-PCP_sta_-X_tei_, X_sta_.* Purification of before mentioned proteins was performed as described by Ulrich et al. (2016) [12].

### Spectral analysis of StaF

StaF was analysed spectroscopically in a concentration of 2.5 µM in 50 mM Tris pH 8.0 at 30 °C using a Jasco V-650 spectrophotometer and the SpectraManager software in order to determine the potentially catalytic active species. Spectral analysis was performed from 390 to 600 nm with 0.2 nm increments from the ferric protein (as purified), the ferrous protein, which had been reduced through the addition of 10 µL of a saturated Na_2_S_2_O_4_ solution, and of the ferrous P450, which had been saturated with CO through bubbling of 60 mL CO gas using a syringe through the cuvette filled with protein solution.

### Protein interaction studies

The interaction analysis of StaF with the A47934 X-domain (X_sta_) was done by analytical size-exclusion chromatography (SEC) using a Superose 12 10/300 GL column connected to an Äkta pure 25 system and the unicorn 6.4 software. The Superose 12 column had been calibrated using Gel Filtration Standard from Bio-Rad (Catalogue number 151-1901) resulting in following elution volumes: 670,000 Da at 8.23 mL, 158,000 Da at 11.27 mL, 44,000 Da at 13.01 mL, 17,000 Da at 14.62 mL for and 1,350 Da at 19.23 mL. 50 mM Tris pH 7.4 and 150 mM NaCl was used as SEC buffer. This method was appropriate for the analysis of the P450 – X-domain interaction as both the protein specific absorption at λ = 280 nm as well as the heme absorption at approximately λ = 415 nm could be monitored. Interaction of StaF and X_sta_ was detected through a significant shift of the heme peak at λ = 415 nm to earlier elution volume when StaF and X_sta_ were analysed together compared to individual analysis of StaF. Prior to analysis, 33.3 µM StaF and 100 µM X_sta_ were incubated at RT for 30 min in SEC buffer in a reaction volume of 100 µL. The reaction was then analysed with the flow rate set to 1 mL/min and detection of the absorption at λ = 280 and 415 nm. Individual analysis of StaF and X_sta_ served as controls.

### P450 activity assay

An in vitro phenolic coupling assay was performed in order to determine the StaF activity. As substrates the teicoplanin-like NH_2_-D-Hpg-D-Tyr-L-Hpg-D-Hpg-D-Hpg-L-Tyr-D/L-Hpg-C(O)R (Tei7(L-Hpg_3_, D/L-Hpg_7_)), the pekiskomycin-like NH_2_-D-Ala-D-Tyr-L-Glu-D-Hpg-D-Hpg-L-Tyr-D/L-Hpg-C(O)R (Pek7(D/L-Hpg_7_)), and the actinoidin-like NH_2_-D-Hpg-D-Tyr-L-Phe-D-Hpg-D-Hpg-L-Tyr-D/L-Hpg-C(O)R (Act7(D/L-Hpg_7_)) heptapeptide were used, which were synthesised according to Brieke et al. [18,36]. It has to be noted that the Hpg-residue at position 7 of all heptapeptides is highly racemisation prone. In case of the Tei7 peptide effective separation by preparative HPLC was possible, so that pure L-Hpg_7_ and D-Hpg_7_ peptide could be used [13]. The diastereomers of Pek7 and Act7 were not separated by preparative HPLC, so that both peptides were used with a racemic mixture of D/L-Hpg_7_ [17]. The activity assay was performed as described in Brieke and Peschke et al. [17] with the first step being the loading of the substrate peptide onto the PCP-X construct (MBP-PCP_sta_-X_sta_, MBP-PCP_tei_-X_tei_, MBP-PCP_tei_-X_sta_, MPB-PCP_sta_-X_tei_) using the R4-4 mutant of the promiscuous phosphopantetheinyl transferase Sfp, subsequently, the actual activity assay was performed using palustrisredoxin B (A105V), palustrisredoxin reductase and NADH as P450 electron source [38], and finally the peptides were purified by solid-phase extraction and analysed HPLC–MS [17]. The StaF activity assays with Tei7-L-Hpg_7_ as substrate were performed both with and without StaH. All other StaF activity assays were always performed together with StaH.

### StaF protein crystallisation

Crystals were grown using hanging drop vapour diffusion at 4 °C. The StaF protein (140 µM) was mixed (1:1) with the reservoir solution (0.1 M phosphate/citrate buffer (pH 4.2), 1.2 M Na_2_PO_4_, 0.3 M K_2_HPO_4_; final pH 5.2) and equilibrated against the reservoir solution. After 10 days red diamonds (≈150 µm length) had formed. The crystals were passed through a cryoprotectant solution (0.1 M phosphate/citrate buffer (pH 4.2), 1.2 M Na_2_PO_4_, 0.3 M K_2_HPO_4_ and either 25% (v/v) glycerol, or 25% (v/v) ethylene glycol) and then flash cooled in liquid nitrogen for data collection. Two native data sets using different cryoprotectant solutions were collected at the X10SA beamline at the Swiss Light Source at the Paul Scherrer Institute (Villigen, Switzerland, λ = 0.9792 Å) with the crystals kept at 100 K during data collection. The data was processed using the XDS program suite [52]. The space group of the crystals was *P*3(1)2(1) with a single P450 molecule per asymmetric unit. The StaF structure was solved using molecular replacement with the program PHASER [53] and a search model consisting of OxyE_tei_ (Protein Data Bank code 3O1A, Chain A) [22], residues 2-384 and heme. Iterative manual model building and refinement were performed using the programs COOT [54] and REFMAC [55] with TLS refinement [56] following a simulated annealing performed in CNS [57–58]. During several rounds of refinement with REFMAC and manual rebuilding, ethylene glycol or glycerol and solvent molecules were included in the models where appropriate. TLS input files were generated using the TLS-Motion Determination Server [59–60]. Structure validation was performed using MOLPROBITY [61] and PROCHECK [62]. Structure-based sequence alignments were carried out with SSM [63] as implemented in COOT and comparisons to known structures performed with DaliLite [64]. All structural figures were prepared using PyMol [65]. Atomic coordinates and structure factor amplitudes have been deposited in the Protein Data Bank (PDB) under accession codes 5EX8 (ethylene glycol cryoprotectant solution) and 5EX9 (glycerol cryoprotectant solution).

## Supporting Information

File 1 HPLC–MS analysis of StaF turnover activity of Tei7-L-Hpg7 (a) and Act7-rac-Hpg7 (b) bound to MBP-PCP-X_tei_.
