# Supplementary material for: Biochemical and structural characterisation of the second oxidative crosslinking step during the biosynthesis of the glycopeptide antibiotic A47934
Source: Beilstein J Org Chem. 2016 Dec 27;12:2849–64. doi: 10.3762/bjoc.12.284 (PMC5238595; doi:10.3762/bjoc.12.284)
Supplement: File 1 — HPLC–MS analysis of StaF turnover activity of Tei7-L-Hpg7 (a) and Act7-rac-Hpg7 (b) bound to MBP-PCP-Xtei. [file Beilstein_J_Org_Chem-12-2849-s001.pdf]

Supporting Information

for

Biochemical and structural characterisation of the second oxidative crosslinking step during the biosynthesis of the glycopeptide antibiotic A47934

Veronika Ulrich[1], Clara Brieke[1] and Max J. Cryle*[1,2,3]

Address: [1]Department of Biomolecular Mechanisms, Max Planck Institute for Medical Research, Jahnstrasse 29, 69120 Heidelberg, Germany [2]EMBL Australia, Monash University, Clayton, Victoria 3800 and [3]The Monash Biomedicine Discovery Institute, Department of Biochemistry and Molecular Biology and ARC Centre of Excellence in Advanced Molecular Imaging, Monash University, Clayton, Victoria 3800.

Email: Max Cryle - max.cryle@monash.edu

*Corresponding author

HPLC–MS analysis of StaF turnover activity of Tei7-L-Hpg7 (a) and Act7-rac-Hpg7 (b) bound to MBP-PCP-X_{tei}

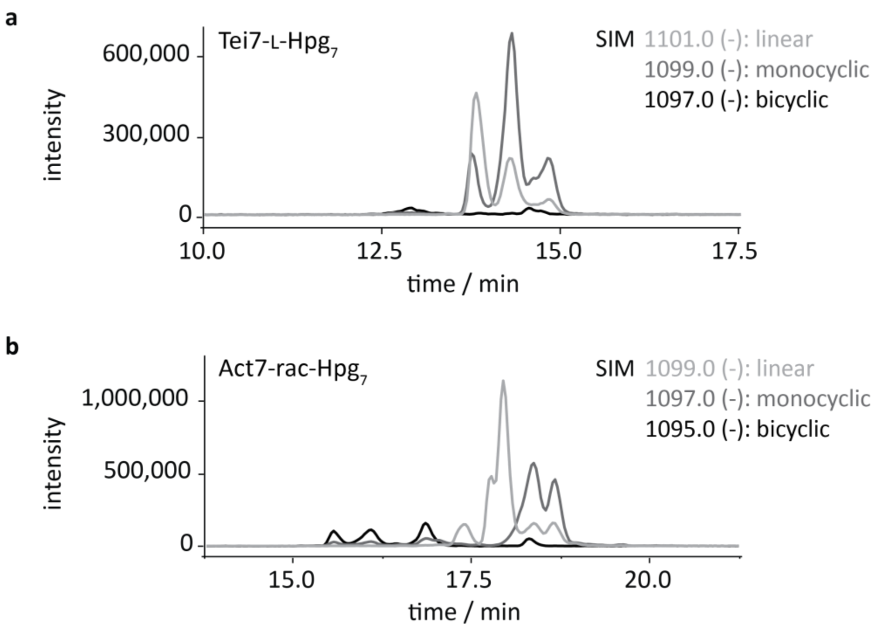

Figure S1: HPLC-MS analysis of StaF turnover activity of Tei7-L-Hpg_7 (a) and Act7-rac-Hpg_7 (b) bound to MBP-PCP-X_{tei}. a) Ions corresponding to singly charged, linear (methylamine *m*/*z* 1101.0; depicted in light grey), monocyclic (methylamine *m*/*z* 1099.0; depicted in grey) and bicyclic Tei7-L-Hpg_7 (methylamine *m*/*z* 1097.0; depicted in black) were recorded using single-ion monitoring (SIM) in negative mode. The minor peak of the monocyclic peptide represents Tei7-D-Hpg_7, which could not be separated completely from Tei7-L-Hpg_7 by preparative HPLC during peptide synthesis. b) Ions corresponding to singly charged, linear (methylamine *m*/*z* 1099.0; depicted in light grey), monocyclic (methylamine *m*/*z* 1097.0; depicted in grey) and bicyclic Act7-rac-Hpg_7 (methylamine *m*/*z* 1095.0; depicted in black) were recorded using single-ion monitoring (SIM) in negative mode. Major peaks for each *m*/*z* represent diastereomers due to racemisation of Hpg_7. a) and b) Smaller peaks are caused through overlapping mass signal detection.
